# The role of microRNAs in defining LSECs cellular identity and in regulating *F8* gene expression

**DOI:** 10.3389/fgene.2024.1302685

**Published:** 2024-02-19

**Authors:** Muhammad Ahmer Jamil, Rawya Al-Rifai, Nicole Nuesgen, Janine Altmüller, Johannes Oldenburg, Osman El-Maarri

**Affiliations:** ^1^ Institute of Experimental Hematology and Transfusion Medicine, University Hospital Bonn, Bonn, Germany; ^2^ Cologne Center for Genomics (CCG), Faculty of Medicine, University Hospital Cologne, University of Cologne, Cologne, Germany

**Keywords:** LSECs, F8, microRNAs profiling, expression, endothelial cells (ECs)

## Abstract

**Introduction:** Coagulation Factor VIII (FVIII) plays a pivotal role in the coagulation cascade, and deficiencies in its levels, as seen in Hemophilia A, can lead to significant health implications. Liver sinusoidal endothelial cells (LSECs) are the main producers and contributors of FVIII in blood, a fact we have previously elucidated through mRNA expression profiling when comparing these cells to other endothelial cell types.

**Methods:** Our current investigation focuses on small microRNAs, analyzing their distinct expression patterns across various endothelial cells and hepatocytes.

**Results:** The outcome of this exploration underscores the discernible microRNAs expression differences that set LSECs apart from both hepatocytes (193 microRNAs at *p* < 0.05) and other endothelial cells (72 microRNAs at *p* < 0.05). Notably, the 134 and 35 overexpressed microRNAs in LSECs compared to hepatocytes and other endothelial cells, respectively, shed light on the unique functions of LSECs in the liver.

**Discussion:** Our investigation identified a panel of 10 microRNAs (miR-429, miR-200b-3p, miR-200a-3p, miR-216b-5p, miR-1185-5p, miR-19b-3p, miR-192-5p, miR-122-5p, miR-30c-2-3p, and miR-30a-5p) that distinctly define LSEC identity. Furthermore, our scrutiny extended to microRNAs implicated in *F8* regulation, revealing a subset (miR-122-5p, miR-214-3p, miR-204-3p, and miR-2682-5p) whose expression intricately correlates with *F8* expression within LSECs. This microRNA cohort emerges as a crucial modulator of *F8*, both directly through suppression and indirect effects on established *F8*-related transcription factors. The above microRNAs emerged as potential targets for innovative therapies in Hemophilia A patients.

## 1 Introduction

Endothelial cells (ECs) are typically found as a single-cell layer lining the inner walls of blood vessels. However, they are not confined solely to the vascular system; they can also be found in the lymphatic system, lining inner channels and tubes, as well as body cavities. Despite sharing some fundamental similarities, these cells exhibit distinct characteristics specific to various tissues and organs. For instance, liver sinusoidal endothelial cells (LSECs) are known as the primary producers of coagulation Factor VIII (FVIII) (Do et al., 1999; Shahani et al., 2010; Everett et al., 2014; Shahani et al., 2014). FVIII is a glycoprotein and irregular expression of coagulation FVIII causes chronic liver disease, similarly in liver cirrhosis elevated levels of FVIII were observed (Pradhan-Sundd et al., 2021). FVIII plays a critical role in the coagulation cascade, and its deficiency, or low levels, results in a bleeding disorder known as Hemophilia A (HA) (Oldenburg and El-Maarri, 2006). Hemophilia A can be categorized as mild (FVIII 6% < FVIII < 49%), moderate (FVIII 1% < FVIII < 6%), or severe (FVIII < 1%) (Oldenburg and El-Maarri, 2006). The current therapeutic approach for HA involves on-demand treatment or prophylaxis, primarily through intravenous injections of recombinant FVIII or the use of bispecific antibodies designed to mimic the interaction between FIX and FX, as seen with Hemlibra from Genentech and Chugai (Kitazawa et al., 2012; Sampei et al., 2013). Furthermore, AAV-based gene therapy has received market approval from both the European Medicines Agency (EMA) and the Food and Drug Administration (FDA) (Rangarajan et al., 2017; Peyvandi and Garagiola, 2019). In addition to vector-based gene therapy, cellular therapy represents another promising avenue for treating hemophilia. This approach involves introducing cells capable of secreting and releasing FVIII into the bloodstream.

However, for cellular and gene therapy to achieve success, it is crucial to gain a deep understanding of the physiology of the cells responsible for producing FVIII, as well as the mechanisms governing its expression and secretion. A pivotal group of molecules contributing to the regulation of mRNA levels within cells is microRNAs, which are small, non-coding RNA molecules composed of 21 nucleotides. They are known to exert control over approximately 30% of all protein-coding genes (Filipowicz et al., 2008). In previous rat studies, certain microRNAs (such as miR-122-5p, miR-126a-3p, miR-335, miR-21-5p, and miR-511-3p) were identified as specific to LSECs (liver sinusoidal endothelial cells) (Oda et al., 2018; Takeuchi et al., 2018). However, for humans, such specific information remains unavailable. Additionally, the potential involvement of microRNAs in the underlying molecular mechanisms of hemophilia A was previously suggested by Jankowska K et al. in 2020 (Jankowska et al., 2020a; Jankowska et al., 2020b).

Our previous mRNA profiling has unveiled remarkable expression markers capable of distinguishing LSECs from both ECs and hepatocytes (El-Maarri et al., 2020; Jamil et al., 2020). Additionally, various research groups have conducted studies to scrutinize the expression profiles of LSECs and compare them with those of other liver cell types, underscoring the unique nature of these cells compared to other resident liver cells (Shahani et al., 2014; MacParland et al., 2018; Aizarani et al., 2019). However, microRNAs studies pertaining to the development and regulation of LSECs have not been comprehensively explored. MicroRNAs analyses have primarily focused on comparisons between different organs or distinct endothelial cell types (Liang et al., 2007; McCall et al., 2011), with limited attention given to LSECs specifically. These previous investigations have illuminated the potential of microRNAs as markers for discerning tissue diversity and distinguishing between various endothelial cell populations (Liang et al., 2007; McCall et al., 2011). Consequently, in our present study, we have performed a comprehensive microRNAs expression profiling comparison between LSECs and other ECs (Oda et al., 2018; Takeuchi et al., 2018), (not expressing much F8, other ECs including HPAEC = Human Pulmonary Arterial ECs, HPMEC = Human Pulmonary Microvascular ECs, and HCMEC = Human Cardiac Microvascular ECs, from here on all occurrence of other ECs correspond to the same 3 ECs we analyzed). This endeavor has opened up new horizons in the field of LSEC characterization, providing invaluable insights into the post-transcriptional regulation of LSECs in relation to other ECs. Our results have not only identified a subset of microRNAs specific to LSECs but have also shed light on their role in the regulation of *F8* gene expression.

## 2 Results

### 2.1 MicroRNAs expression reveals unique signatures in liver sinusoidal endothelial cells (LSECs) distinguishing them from other endothelial cells (ECs) and hepatocytes

**FIGURE 1:**
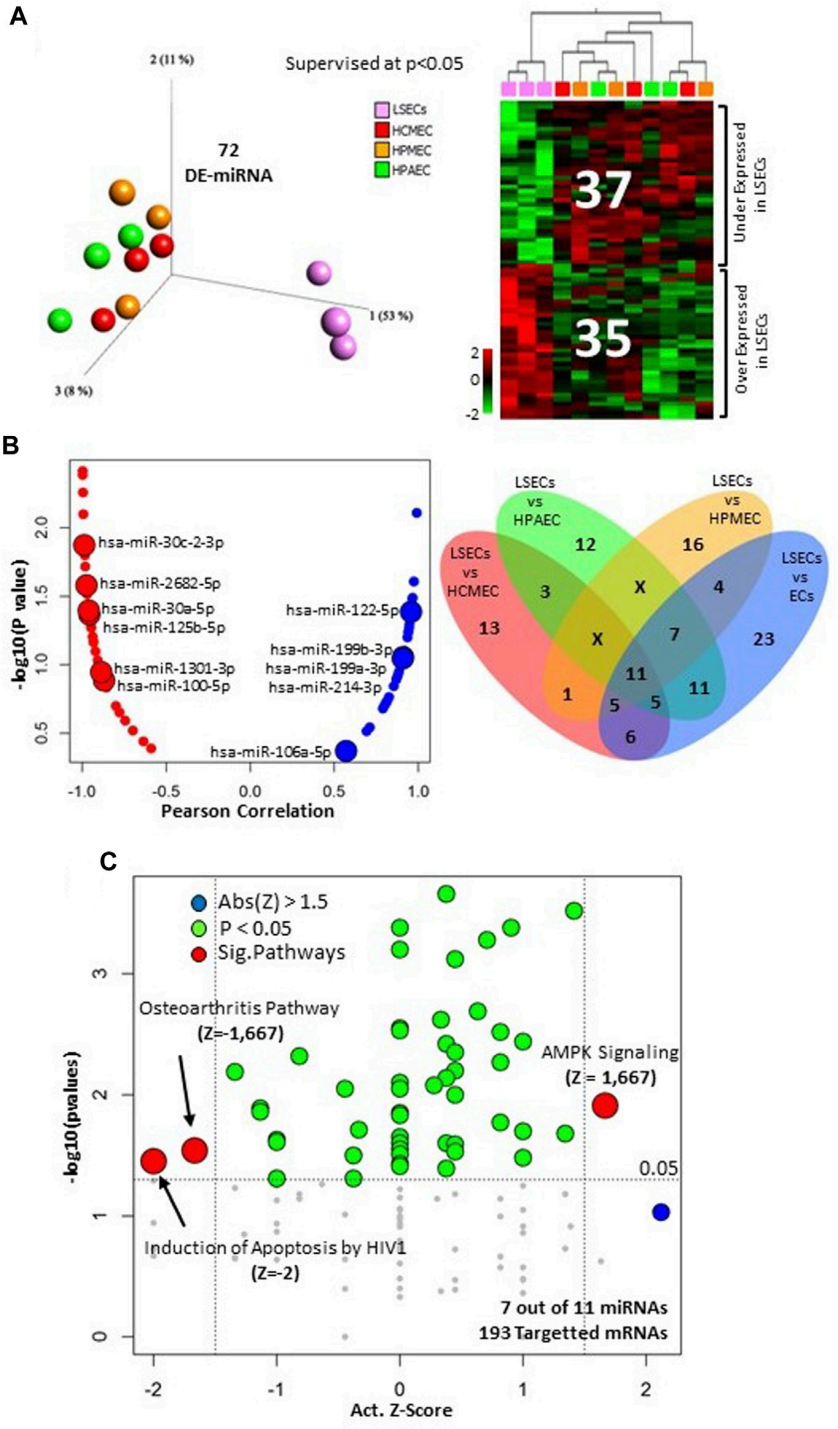
MicroRNA analysis in LSECs. **(A)** Differentially expressed microRNA between LSECs and other ECs at *p* < 0.05. Left = 3D-PCA of differentially expressed microRNAs, Right = Heatmap illustrating the differential expression of microRNAs between LSECs and other ECs. **(B)** Left panel: correlation plot between *F8* expression and microRNA (Blue = Positive Correlated, Red. = Negative Correlated) the 11 microRNAs differentially expressed between LSECs and all other ECs are highlighted; Right panel: Venn diagram of different expression comparison of microRNA in LSECs compared to other ECs at *p* < 0.05. **(C)** Significant canonical pathways of targeted mRNA of the 11 common microRNAs from section **(B)**.

In our prior study, we demonstrated distinct expression patterns in liver sinusoidal endothelial cells (LSECs) when compared to other endothelial cells (Jamil et al., 2020). However, microRNAs were not previously analyzed in human LSECs. In our current investigation, we identified 72 out of 411 microRNAs that displayed significant differential expression (*p* < 0.05) between LSECs and other endothelial cells. Among these, 35 were found to be overexpressed, while 37 were under-expressed in LSECs compared to other endothelial cells (Figure 1A, Supplementary Table S1).

Upon comparing LSECs with individual endothelial cell types (HCMEC, HPAEC, and HPMEC), we identified several differentially expressed microRNAs. Notably, 11 microRNAs were found to be commonly differentially expressed in all three comparisons (LSECs-HPAEC, LSECs-HPMEC, LSECs-HCMEC) (Figure 1B Right, Supplementary Table S2). Furthermore, seven of these microRNAs were observed to target 427 out of 2,547 differentially expressed genes (DEGs) identified in our previous study comparing LSECs and other endothelial cells at *p* < 0.05 (Supplementary Table S3) (Jamil et al., 2020). The correlation analysis of these 11 microRNAs with *F8* expression revealed a strong negative correlation for “miR-30c-2-3p” and a significantly positive correlation with “miR-122-5p” (Figure 1B Left). Pathway analysis of the 427-targeted genes (over/under expressed) indicated decreased activity in pathways related to “Induction of Apoptosis by HIV1” and “Osteoarthritis,” whereas the “AMPK Signaling” pathway displayed significant activation (Figure 1C).

**FIGURE 2:**
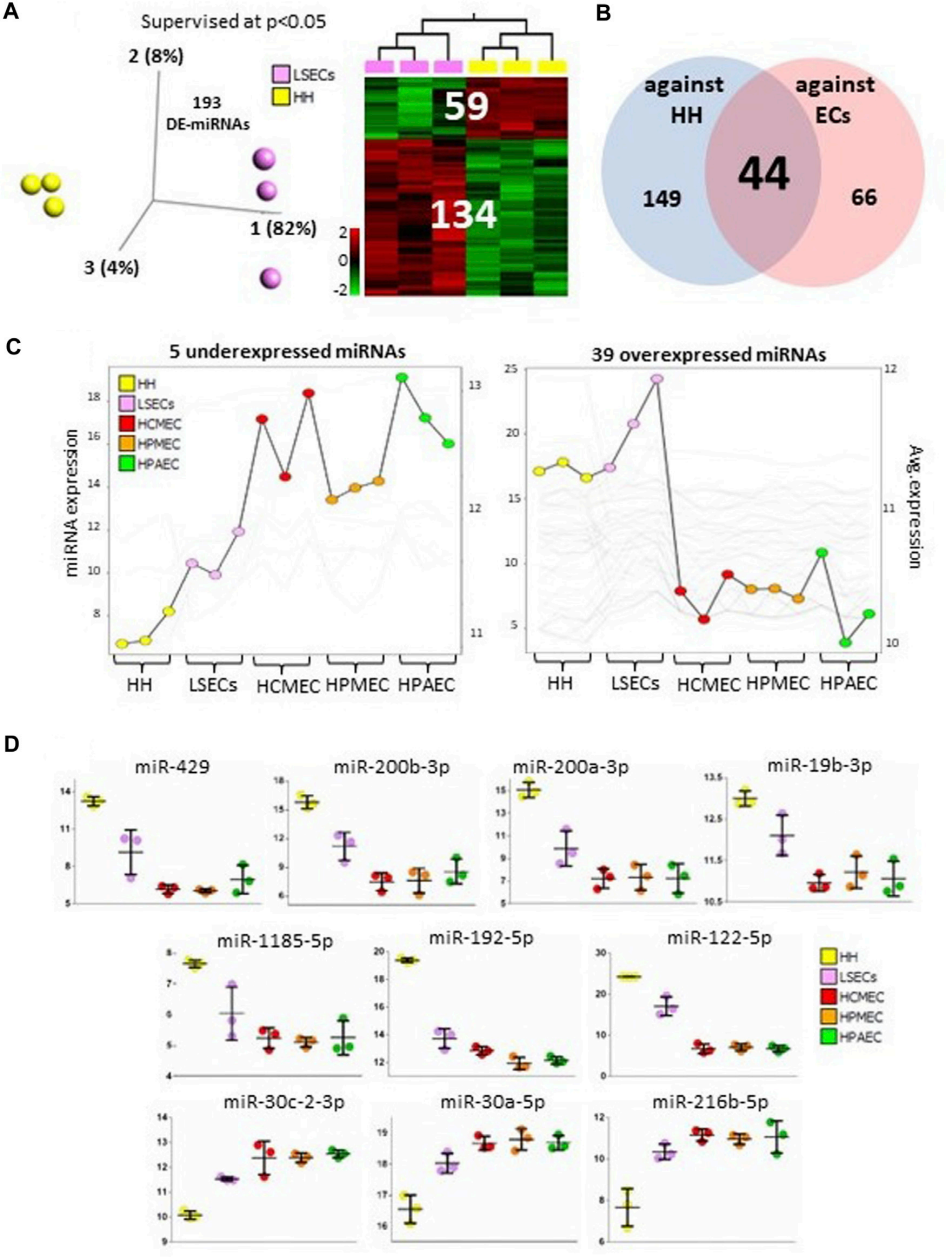
MicroRNAs in LSECs vs. HH. **(A)** 3D-PCA and Heatmap plots illustrating differentially expressed microRNAs between LSECs and hepatocytes at *p* < 0.05. **(B)** Venn diagram displaying differentially expressed microRNAs between LSECs vs hepatocytes and LSECs vs. other endothelial cells. **(C)** Summary graphs of expression of differentially expressed between both LSECs vs. hepatocytes and LSECs vs. other endothelial cells in all samples (Left panel: five under expressed microRNAs; Right panel: 39 over-expressed microRNAs). **(D)** Dot plot representing data from all samples and highlighting the top 10 significant microRNAs.

Hepatocytes constitute 55%–65% of liver mass. Comparing hepatocytes (HH) with LSECs can offer insights into the specific microRNAs that define LSEC identity. We identified 193 differentially expressed microRNAs (134 upregulated and 59 downregulated in LSECs) between LSECs and HH at *p* < 0.05 (Figure 2A, Supplementary Table S4). Among these, 44 microRNAs (39 upregulated and 5 downregulated in LSECs compared to other endothelial cells) were common with differentially expressed microRNAs found when comparing LSECs with hepatocytes and other endothelial cells (Figure 2B). Overexpressed microRNAs (39 microRNAs) exhibited the highest expression in LSECs when compared to both hepatocytes and other endothelial cells (Figure 2C Right), while the mean expression of under-expressed microRNAs (5 microRNAs) in LSECs fell between that of hepatocytes and other endothelial cells (Figure 2C Left). The top 10 differentially expressed microRNAs in LSECs compared to hepatocytes and other endothelial cells included miR-122-5p, miR-192-5p (liver-specific microRNAs) (Chang et al., 2004; Liang et al., 2007; Coll et al., 2015), miR-19b-3p, miR-30c-2-3p (F8/HA associated microRNAs) (Jankowska et al., 2020a; Atreya et al., 2020), and several other microRNAs (miR-429, miR-200b-3p, miR-1185-5p, miR-200a-3p, miR-30a-5p, and miR-216b-5p) (Figure 2D).

### 2.2 Distinctive expression patterns of the top 100 microRNAs in LSECs set them apart from other endothelial cells

**FIGURE 3:**
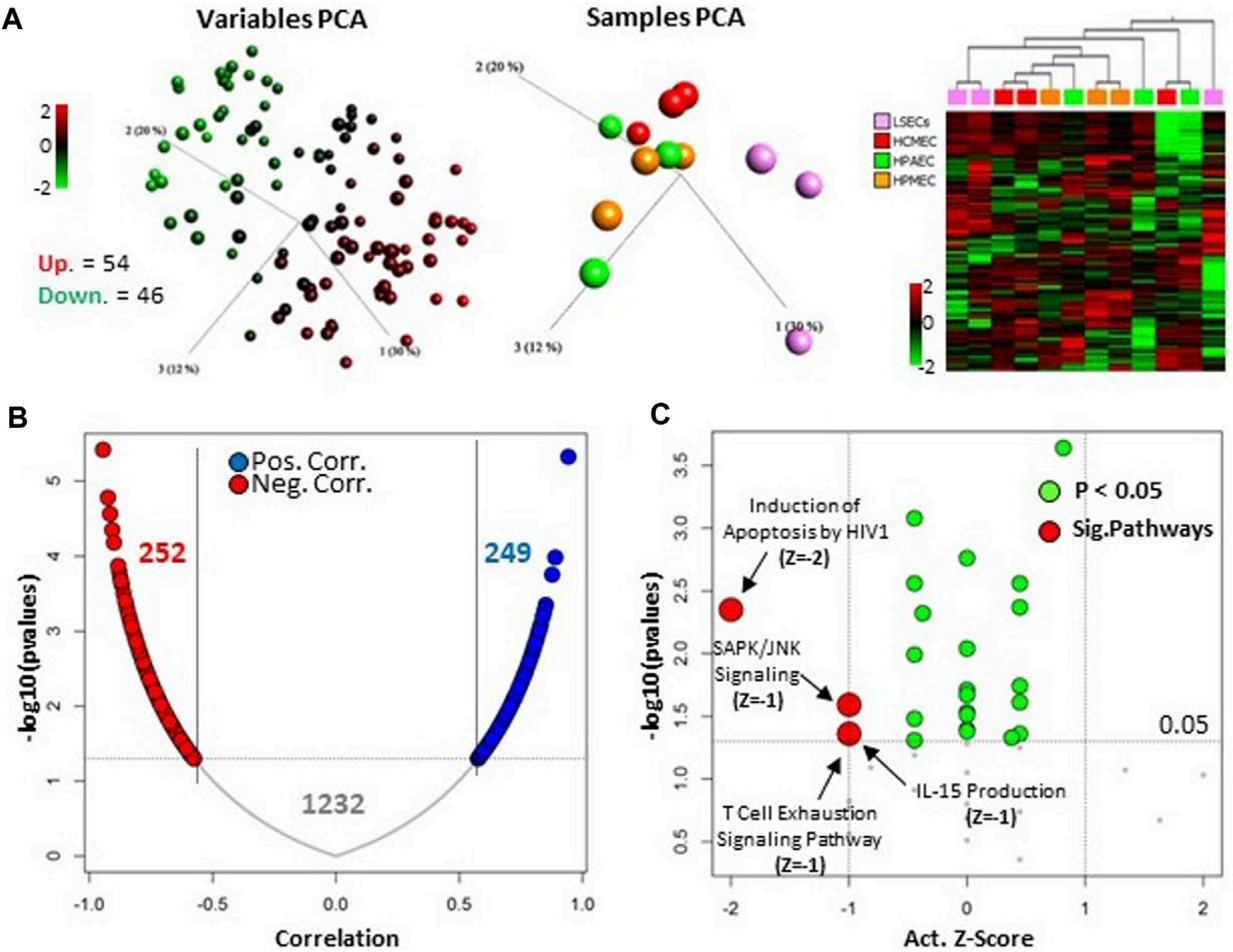
Upper 100 expressed microRNA in LSECs. **(A)** Left panel: Unsupervised, 3D variable PCA of individual top 100 microRNAs in LSECs showing overexpression as red and under-expression as green in LSECs compared to other ECs. Middle panel: Unsupervised, 3D sample PCA based on upper expressed 100 microRNAs in LSECs. Right panel: Heatmap of top 100 microRNAs expressed in LSECs. **(B)** Correlation of top 100-expressed microRNAs in LSECs targeting differentially expressed mRNA between LSECs and other ECs from our previous study (Jamil et al., 2020, licensed CC-BY-4.0). **(C)** Significant canonical pathways associated with the 252 negatively correlated targeted mRNA of the top 100 microRNA expressed in LSECs.

The top 100 microRNAs expressed in LSECs consist of 54 upregulated and 46 downregulated microRNAs when compared to other endothelial cells (ECs) (Figure 3A, Supplementary Table S5). Intriguingly, 42 out of these top 100 microRNAs were also identified among the top 100 expressed microRNAs in a study by Oda et al. in 2017, which focused on rat LSECs samples (GSE97229) (Oda et al., 2018) (Supplementary Tables S5, S6).

Moreover, we observed that 75 out of these 100 top microRNAs target a substantial number of differentially expressed genes (DEGs), specifically 1,293 out of 2,547, as identified in our prior study comparing LSECs and other ECs (Jamil et al., 2020). Remarkably, the expression of these microRNAs exhibited a significantly negative correlation with the expression of only 252 genes (Figure 3B). Pathway analysis of these negative correlated-targeted genes highlighted the enrichment of specific signaling pathways, notably “SAPK/JNK Signaling” and the “T-Cell Exhaustion Signaling Pathway” (Figure 3C). Additionally, other significant pathways identified included “Induction of Apoptosis by HIV1” and “IL-15 Production.” These pathways are linked to an immune response which is known functionality for LSECs (Jamil et al., 2020). Similarly, stress or apoptosis-related pathways are linked to chronic liver diseases which are related to irregular FVIII expression (Pradhan-Sundd et al., 2021).

### 2.3 MicroRNAs targeting *F8*-mRNA also contribute to LSECs identity

**FIGURE 4:**
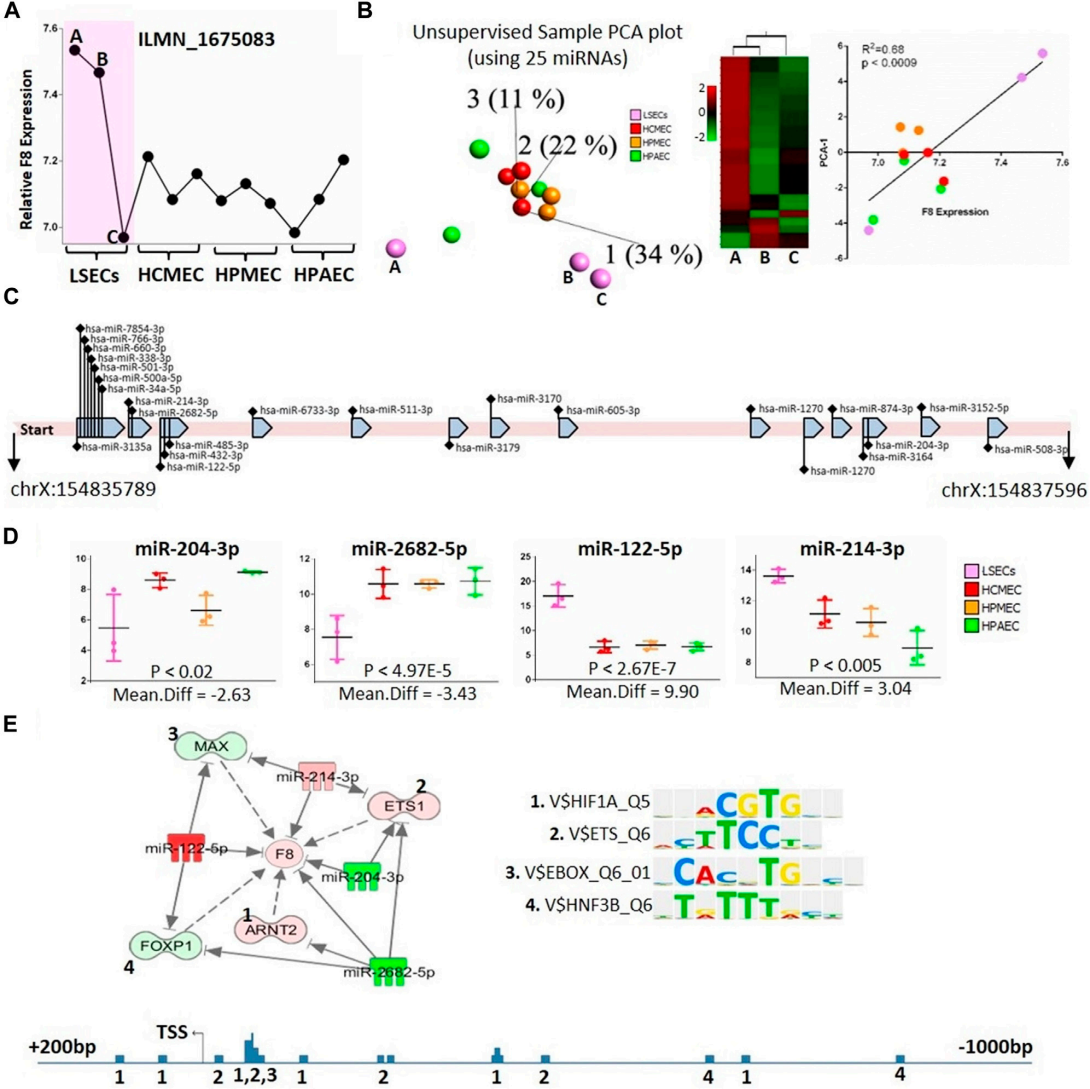
microRNAs targeting *F8* gene. **(A)** Relative expression of *F8* gene (Probe ID: ILMN_1675083) in the used ECs and LSECs samples. **(B)** Left panel: Unsupervised, 3D-PCA displaying different endothelial cells for 25 expressed microRNAs that bind to *F8* gene according to IPA. Middle panel: heat map showing the expression of these 25 microRNAs in three LSEC samples. Right panel: Correlation between *F8* expression in different endothelial cells and PCA-1, X-axis represents *F8* expression and y-axis represents PCA-1. **(C)** A depiction of the 1808-base pair long 3′ mRNA sequence of the *F8* gene along with the 25 expressed microRNAs known to bind to this sequence. **(D)** Box plots illustrating the expression levels of four microRNAs in different endothelial cells, potentially binding to the *F8* gene, as well as the transcription factors binding to the *F8* gene promoter. **(E)** Top Panel: An IPA-generated figure showing the relationships between potentially *F8*-binding microRNAs and transcription factors identified using TRANSFAC, which bind to the *F8* promoter. Sequence logos of individual transcription factors are displayed on the right. Bottom Panel: Representation of the *F8* promoter, covering 1,200 base pairs with 1 kb before the transcription start site and 200 base pairs after the transcription start site. The numbers represent the transcription factors binding sites identified using TRANSFAC (shown and numbered in top panel above).

Considering that hepatocytes are recognized as non-FVIII-producing cells (Shahani et al., 2014), this section exclusively focuses on the analysis of LSECs and endothelial cells in relation to *F8*-targeting microRNAs. We incorporated all three LSEC samples, notably including two samples (samples B and C) exhibiting relatively high *F8* expression and one sample (sample A) displaying lower *F8* expression (Figures 4A, B).

Our analysis, utilizing the IPA database, identified 106 microRNAs that target *F8*. Out of these, only twenty-five microRNAs were expressed in our microRNA-sequencing data (Figures 4B, C, Supplementary Tables S7, S8), and these microRNAs were capable of distinguishing LSECs from other endothelial cells. A 3D-PCA and heatmap analysis revealed that among the three LSEC samples, one (Sample A) exhibited overexpression of a majority of microRNAs targeting *F8* (Figure 4B Left and Middle, Supplementary Table S7). Notably, this LSEC sample demonstrated significantly lower levels of *F8* expression, even lower than that observed in other ECs (Figure 4A).

Furthermore, we observed a significant positive correlation (*R* ^2^ = 0.68) between the first principal component analysis (PCA) of microRNAs targeting *F8* and the expression of *F8* itself (Figure 4B Right panel). Conducting a differential expression analysis of microRNAs between LSECs and other ECs unveiled four potential microRNAs (miR-204-3p, miR-2682-5p, miR-122-5p, and miR-214-3p) that not only distinctively separated LSECs from other ECs but most probably post-transcriptionally regulated *F8* (Figures 4C, D). Additionally, it was noted that these microRNAs also regulated other transcription factors (TFs), such as ETS1, MAX, ARNT2, and FOXP1, known to bind to the *F8* promoter (Figure 4E).

### 2.4 Selected microRNAs effects on *F8* expression proved in primary LSECs

**FIGURE 5:**
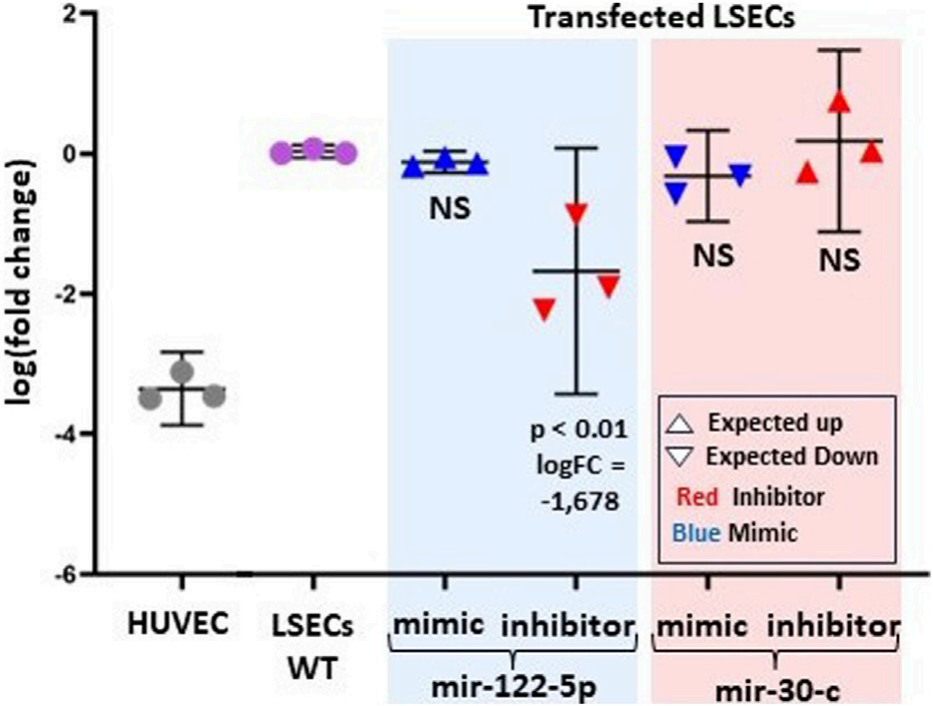
Experimental evidence of the effect of two-selected microRNAs (mir-122-5p and mir-30-c) on *F8* mRNA levels. The log fold change in *F8* expression from RT-PCR is compared to LSECs-WT (Wild Type) and HUVEC (Negative Control). LSECs were transfected with miR-122-5p (both mimic and inhibitor) and miR-30-c (both mimic and inhibitor). In the graph, red represents inhibitors, and blue represents mimic microRNAs. An upward-pointing triangle indicates the expected/hypothesized upregulation, while a downward-pointing triangle indicates the expected/hypothesized downregulation of *F8* in LSECs.

We selected two microRNAs, miR-122-5p (positively correlated) and miR-30-c (negatively correlated) with *F8* expression levels, to assess their impact on primary LSECs. Figure 5 illustrates that both of these selected microRNAs have an effect on F8 expression, albeit to varying degrees. To investigate this, we chose one mimic and one inhibitory molecule for each of the two selected microRNAs.

As anticipated, the miR-122-5p inhibitory molecule significantly reduced *F8* expression in LSECs compared to LSECs-WT (*p* < 0.01, logFC = −1.67). However, we observed a statistically non-significant overexpression of *F8* for the mir-30-c inhibitory molecule. Furthermore, there was a slight, non-significant under-expression observed for both the miR-30-c mimic and miR-122-5p mimic.

## 3 Discussion

LSECs are a distinctive subset of endothelial cells, differing not only in morphology and physiology but also in their anatomical location. While expression profiles of LSECs have been examined in previous studies by various research groups to elucidate their functional characteristics (Shahani et al., 2014; MacParland et al., 2018; Aizarani et al., 2019; El-Maarri et al., 2020; Jamil et al., 2020), a detailed analysis of microRNA expression levels in human LSECs has been lacking. Although microRNA expression in rat LSECs has revealed some microRNAs associated with LSECs (Oda et al., 2018), the overlap with microRNA profiling in human LSECs remained unexplored.

In the rat study, microRNAs specific to LSECs, distinct from hepatocytes, were identified, including miR-122-5p, miR-21-5p, miR-30c-5p, let-7 microRNAs, and others (Oda et al., 2018). Interestingly, these microRNAs were also found in our comparisons of LSECs against other endothelial cells. Among these, miR-122-5p exhibited the highest fold change between LSECs and other endothelial cells. This microRNA is known for its liver specificity, irrespective of the cell type (Liang et al., 2007), suggesting that its overexpression might be pivotal for defining the liver cell identity of LSECs. Similarly, miR-214, the second-highest differentiated microRNA in our analysis between LSECs and other endothelial cells, has been linked to liver fibrogenesis. In fact, the knockout of miR-214 has shown promise as a therapy for liver fibrosis (Ma et al., 2018). Therefore, liver-specific markers like miR-122-5p and miR-214 might be present in all liver organ cells, including LSECs.

Other microRNAs overexpressed in LSECs may be shared across various organs. For instance, miR-199a-5p and miR-199b-5p/3p, overexpressed in LSECs, have also been previously identified as overexpressed in arterial ECs (McCall et al., 2011). Moreover, both miR-199a and miR-199b are overexpressed in hematological cells (McCall et al., 2011). The biological functions associated with specific microRNAs can also be shared across different cell types. In this context, we identified miR-106a-5p and miR-98-5p as differentially expressed microRNAs with overexpression in LSECs compared to other endothelial cells. These microRNAs are known to suppress proliferation in different cell types (He et al., 2016; Hu et al., 2018), which could explain the lower proliferation rate of LSECs in comparison to other endothelial cells. Additionally, miR-30a targets genes known to regulate liver development and function (Hand et al., 2009), while miR-20a is a known regulator of tip cell formation in endothelial cells (Jiang et al., 2013). The downregulation of these microRNAs in LSECs aligns with the maturation of LSECs, which occurs between 5 and 12 weeks of gestation (Poisson et al., 2017). The specific functional effects of LSECs-specific microRNAs are further supported by the fact that genes targeted by the top expressed microRNAs in LSECs are enriched in pathways previously identified to play a role in LSECs characterization (Jamil et al., 2020).

The liver consists of multiple cell types, with hepatocytes comprising the majority, while non-parenchymal cells, including LSECs, make up only 6.5% of the total liver volume (Trefts et al., 2017). MicroRNA profiling of LSECs, showing differential expression compared to both hepatocytes and other endothelial cells, allows us to categorize and identify cells based on both their host organ and cell type. In this regard, we identified/distinguished three sets of microRNAs: **A)** Liver-specific microRNAs (miR-192-5p, miR122-5p, miR-30c-2-3p, and miR-30a-5p), as discussed earlier. **B)** LSECs-specific microRNA: miR-200 family (miR-429, miR-200b-3p, and miR-200a-3p) distinguished LSECs from both hepatocytes and other endothelial cells. The expression pattern of the miR-200 family showed continuous expression from other endothelial cells to LSECs, ultimately reaching high expression levels in hepatocytes. These microRNAs are known to play a role in the differentiation of human embryonic stem cells (hESCs) into hepatocytes by gradually decreasing the expression level of the targeted ZEB1 gene (Kim et al., 2017). Although the miR-200 family is not significantly expressed in endothelial cells, their higher expression in LSECs compared to other endothelial cells underscores the classic fact that endothelial cells possess host organ features. **C)** Endothelial cells-specific microRNAs: miR-216b-5p, which was downregulated in both LSECs and hepatocytes, has SOX9 as a major target (Liu et al., 2018). SOX9 is known to play a crucial role in liver organogenesis (Kawaguchi, 2013). Additionally, miR-1185-5p, upregulated in both LSECs and hepatocytes, is a key regulator of the mTOR signaling pathway (Alqurashi et al., 2019), which we previously found to be predicted as inhibited in LSECs (Jamil et al., 2020).

LSECs are the primary endothelial cells expressing and producing plasma circulating FVIII, a finding confirmed by our expression data (El-Maarri et al., 2020; Jamil et al., 2020). It was shown that different endothelial cells, i.e., lymphatic endothelial cells or glomerular endothelial cells express F8 (Pan et al., 2016) but an increment in levels of F8 was observed in hemophilia patients after liver transplantation leading to the fact that the main contributor of plasma F8 is the liver (Marchioro et al., 1969; Bontempo et al., 1987). We observed that one LSEC sample (Sample A) exhibited low levels of *F8* expression compared to Samples B and C (Figure 4A). Interestingly, microRNAs targeting *F8* in the low-level *F8* LSECs (Sample A) were upregulated compared to the high-level *F8* LSECs (Samples B and C). It showed the fact that LSECs are of different types (Strauss et al., 2017) and not all LSECs have a similar amount of F8 expression (Jamil and El-Maarri, 2020). Also, the commercial source of our cell line does not provide the information about the sampling region of the LSECs which may change the type of LSECs based on the region like central venous, or periportal. Meanwhile, the first principal component analysis (PCA) of the 3D-PCA plot of microRNAs expression targeting *F8* in all endothelial cells showed a correlation with *F8* expression. This highlights the critical role of microRNAs targeting *F8* in the expression of *F8* in LSECs. This is further supported by the fact that the differential gene expression analysis of *F8*-targeting microRNAs identified four significant microRNAs (miR-204-3p, miR-2682-5p, miR-122-5p, and miR-214-3p) between LSECs and other endothelial cells (Figure 4E). The downregulation of miR-2682-5p and miR-204-3p upregulates the key transcription factor of *F8*, ETS-1, while miR-214-3p and miR-122-5p may control *F8* expression by suppressing transcription through the targeting of MAX and FOXP1 transcription factors (Figure 4E). We validated two microRNAs through *in-vitro* experiments using primary LSECs and found that the inhibitors corresponding to miR-122-5p and miR-30-c decreased and increased *F8* mRNA expression, respectively (Figure 5).

In summary, we have conducted the first characterization of the microRNAs expression profile of isolated human primary LSECs. We have identified LSEC-specific microRNAs that are either shared with the host liver organ or unique to LSECs. These LSEC-specific microRNAs play a role in regulating the *F8* gene expression.

## 4 Materials and methods

### 4.1 Materials

#### 4.1.1 MicroRNAs expression and RNA-seq analysis material

Cellular materials of small RNAs, were procured from ScienCell Research Laboratories located at Corte Del Cedro, Carlsbad, CA 92011, through Provitro GmbH at Chariteplatz 1, 10117 Berlin. These materials were sourced from three different male donors. Notably, all molecular materials (cells, RNA, and DNA) were obtained at passage one, as indicated in the manufacturing certificate. ScienCell Research Laboratories provide an ethical statement regarding the isolation of the samples, which can be accessed on their website at: https://www.sciencellonline.com/ethical-statement. Recommended culture media from ScienCell is endothelial culture medium for all endothelial cells bought. Furthermore, the ethics committee of the University Clinic-Bonn granted approval for the utilization of these samples, with an approval number of 041/13. The quality assessment of LSECs was conducted in our prior study, where we established the similarities between our LSECs and single-cell data (Oda et al., 2018). Epigenetic and expression profiling of hepatocytes and endothelial cells were previously reported in our research, with an accession number GSE140079 (El-Maarri et al., 2020; Jamil et al., 2020).

#### 4.1.2 Experimental validation material

In this study, Primary adult Human Liver Sinusoidal Endothelial Cells (LSECs) were procured from Biozol Diagnostica Vertrieb GmbH (product lot No. HEC03020491) and cultured in a specialized endothelial cell culture medium, denoted as ENDO-Growth Medium MED001, supplemented with growth factors and antibiotics. The cell cultures were maintained at a temperature of 37°C in a humidified atmosphere containing 5% CO_2_ to ensure optimal cell growth and viability. The microRNAs of interest, including both mimic and inhibitor types, were sourced from Qiagen. Before transfection, these microRNAs were diluted in nuclease-free water to achieve the desired concentrations, specifically 5 nM for the mimic microRNA and 50 nM for the microRNA inhibitor. For the transfection process, primary adult LSECs were seeded into 48-well plates at a density that would result in approximately 70%–80% confluency at the time of transfection. To minimize the influence of serum components, the growth medium was replaced with a serum-free culture medium 1–2 h before transfection. Transfection complexes were prepared by gently mixing 100 μL of the diluted microRNA with 3 μL of HiPerFect transfection reagent, following the manufacturer’s protocol. The mixture was then incubated at room temperature for 10 min to allow for complex formation. Subsequently, the transfection complexes were added dropwise to the cells, and the plates were gently swirled to ensure a uniform distribution of the transfection reagent throughout the culture. The cells were then incubated with the transfection complexes for 48 h while maintaining a temperature of 37°C in a humidified atmosphere with 5% CO_2_.

### 4.2 RT-PCR analysis

After 48 h following transfection, we evaluated the functional impact of the miRNA on primary LSECs by conducting quantitative real-time PCR (QRT-PCR) to assess the expression levels of targeted genes. To perform this analysis, we extracted total RNA from the transfected cells using the Invitrogen PureLink RNA Mini Kit (Product Code: 10359103). Subsequently, we used 10 ng of total RNA for a one-step RT-PCR assay to measure *F8* expression using an ABI 7500 real-time PCR system. Specific primers for *F8* (Hs00252034_m1, Thermo Fisher, Catalog No. 4331182) were utilized, along with β-actin primers as an internal control. The amplifications were carried out utilizing 25x RT-PCR Master Mix (AgPath-ID™ One-Step RT-PCR Reagents, Applied Biosystems by Thermo Fisher) and 0.6 pmol of each primer. The PCR protocol involved an initial step at 95 °C for 5 min, followed by 40 cycles of denaturation at 95 °C for 30 s, annealing at 60 °C for 30 s, extension at 72 °C for 45 s, and a final extension step at 72 °C for 7 min. To ensure the reliability and reproducibility of our findings, we conducted all experiments in triplicate, with biological triplicates being included. Ct-values from the RT-PCR were transformed into fold changes using the 2^−ΔΔCT^ method. Subsequently, the data underwent statistical analysis using appropriate tests, and the results were presented as the mean ± confidence interval (CI).

Primers:

Beta-Actin-F: ACC​TTC​TAC​AAT​GAG​CTG​CG,

Beta-Actin-R: CCT​GGA​TAG​CAA​CGT​ACA​TGG,

Probe Beta-Actin: JOE-ACCTGGGTCATCTTCTCGCGGTTG-BHQ1.

### 4.3 microRNAs analysis

The microRNAs were prepared and sequenced using the “TruSeq Small RNA Library Preparation Kits | Sequence micro and small RNAs” kit from Illumina (Prep Kit Code: RS-200-0012). The quality of the sequence data was assessed using the “fastq” software. To ensure data integrity, we utilized Cutadapt for the trimming of adapter sequences and removal of low-quality reads. For mapping the sequences, we used the “miARMA-seq” pipeline on the GRCh38 reference genome. The mature microRNAs GFF file necessary for feature count analysis was obtained from Ensemble. To facilitate downstream analysis, the count table generated by miARMA-seq was converted into FPKM (Fragments Per Kilobase Million) using Qlucore Omics Explorer 3.5. In order to focus our analysis on meaningful microRNAs, those with very low read counts across all samples were filtered out prior to conducting differential microRNAs expression analysis. Finally, the assessment of differential microRNA expression was carried out using Qlucore Omics Explorer 3.5, employing a statistically significant threshold of *p* < 0.05. This rigorous approach ensured the robustness and reliability of our microRNAs expression analysis.

### 4.4 microRNAs—mRna target analysis

We used the microRNA target filter function within the Ingenuity Pathway Analysis (IPA) to identify potential microRNA targets. Since microRNAs are well-known negative regulators, we specifically focused on microRNAs-mRNA interactions with a negative correlation or showing negative regulation. The subsequent processing of the microRNAs-mRNA target data was carried out using the R platform, allowing for further in-depth analysis. To identify microRNAs that bind to the *F8* gene, we utilized IPA. We then conducted a sequence match analysis by comparing these microRNAs with the 1808-base pair long 3′ mRNA sequence of *F8*, which we obtained from UCSC. For the microRNA sequences, we sourced the data from miRbase. Pairwise sequence alignment was performed using the EBI Emboss Matcher. This comprehensive approach, (Supplementary Table S8), allowed us to explore potential microRNAs interactions with the *F8* gene and further our understanding of the regulatory mechanisms involved.

### 4.5 Pathway analysis

Pathway analysis was conducted using Ingenuity Pathway Analysis (IPA). To perform this analysis, we entered the differentially expressed genes into IPA, utilizing the mean difference for enrichment analysis and the calculation of activity Z-Score. This approach allowed us to gain valuable insights into the functional pathways and their regulatory activity associated with the observed gene expression changes.

### 4.6 Transcription factor analysis

Transcription factor was identified using TRANSFAC database. *F8* promoter of 1,000 bp upstream and 200 bp downstream was entered into TRANSFAC match tool with a profile selected for vertebrate_non_redundant_minSum and data version 2020.2. Transcription factor and microRNAs interaction was identified using IPA analysis.

We identified potential transcription factors by utilizing the TRANSFAC database. Specifically, we analyzed the *F8* promoter region, encompassing 1,000 base pairs upstream and 200 base pairs downstream of the *F8* gene. This sequence was entered into the TRANSFAC match tool with a profile selected for vertebrate_non_redundant_minSum and using data from version 2020.2 of the database.

Furthermore, we conducted an analysis of the interactions between transcription factors and microRNAs using Ingenuity Pathway Analysis (IPA). This comprehensive approach allowed us to explore the regulatory networks involving transcription factors and microRNAs, providing insights into the intricate regulatory mechanisms associated with the *F8* gene and related processes.

### 4.7 Statistical analyses

All statistical analyses were conducted within the statistical environment of R or GraphPad Prism 6. Specifically, for the generation of 3D-PCA (Principal Component Analysis) and heatmaps, we utilized Qlucore Omics Explorer 3.5. For other types of plots and data visualization, we used either R or GraphPad Prism 6.

## Data Availability

The datasets presented in this study can be found in online repositories. The names of the repository/repositories and accession number(s) can be found below: https://www.ncbi.nlm.nih.gov/geo/, GSE242926; https://www.ncbi.nlm.nih.gov/geo/, GSE140079.
