# Supplementary material for: The role of microRNAs in defining LSECs cellular identity and in regulating F8 gene expression
Source: Front Genet. 2024 Feb 19;15:1302685. doi: 10.3389/fgene.2024.1302685 (PMC10910020; doi:10.3389/fgene.2024.1302685)
Supplement: Supplementary file 2 [file DataSheet1.docx]

Supplementary Material

# Supplementary Figures and Tables


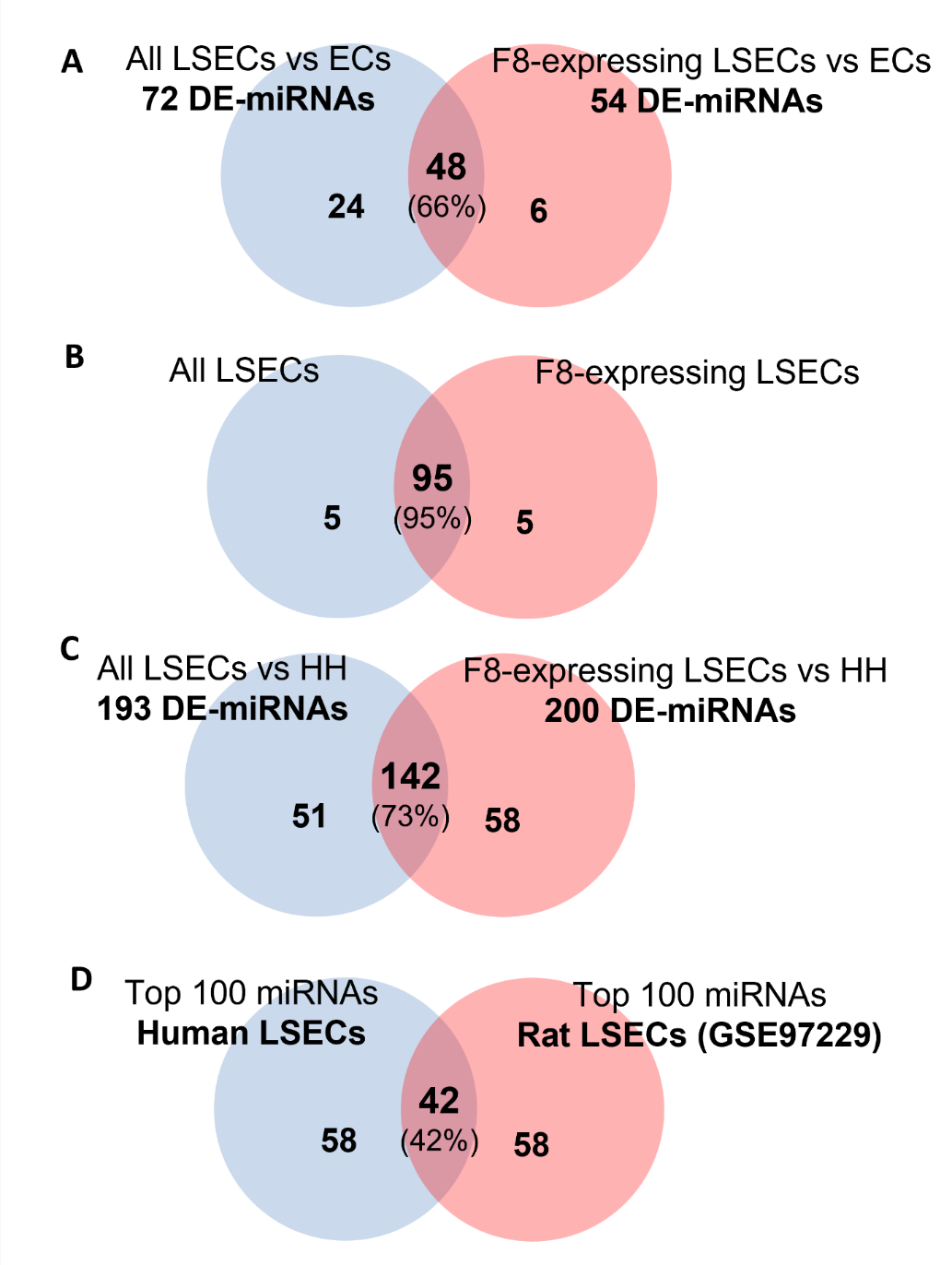


**Supplementary Fig. 1.** Venn Analysis of **A)** Differentially expressed miRNAs in All LSECs vs ECs with differentially expressed miRNAs in F8-expressing LSECs vs ECs, **B)** Top 100 expressed miRNA in All LSECs with top 100 expressed miRNAs in F8-expressing LSECs, **C)** Differentially expressed miRNAs in All LSECs vs Hepatocytes with differentially expressed miRNAs in F8-expressing LSECs vs Hepatocytes, **D)** Top 100 miRNAs expressed in human LSECs with Top 100 miRNAs expressed in Rat LSECs (GSE97229)[9].

**Supplementary Table 1.** Differentially expressed miRNAs (72 miRNAs) between LSECs and other ECs at p < 0.05

**Supplementary Table 2.** Venn analysis of one to one comparisons of LSECs with other ECs (HPAEC, HPMEC, and HCMEC) and all ECs combined

**Supplementary Table 3.** Common miRNAs from Fig. 1B targeting DEGs from our previous study [11].

**Supplementary Table 4.** Differentially expressed miRNAs (193 miRNAs) between LSECs and hepatocytes

**Supplementary Table 5.** Top 100 miRNAs from Rat LSECs (GSE97229) [9] and human LSECs

**Supplementary Table 6.** Venn analysis of top 100 miRNAs in Rat LSECs (GSE97229) [9] with top 100 miRNAs in human LSECs.

**Supplementary Table 7.** Expression in individual LSECs of F8 targeting miRNAs expressed in our dataset.

**Supplementary Table 8.** Pairwise alignment details of miRNA binding to 3’mRNA of F8
